# Comparative Genomics of the Balsaminaceae Sister Genera *Hydrocera triflora* and *Impatiens pinfanensis*

**DOI:** 10.3390/ijms19010319

**Published:** 2018-01-23

**Authors:** Zhi-Zhong Li, Josphat K. Saina, Andrew W. Gichira, Cornelius M. Kyalo, Qing-Feng Wang, Jin-Ming Chen

**Affiliations:** 1 Key Laboratory of Aquatic Botany and Watershed Ecology, Wuhan Botanical Garden, Chinese Academy of Sciences, Wuhan 430074, China; wbg_georgelee@163.com (Z.-Z.L.); jksaina@wbgcas.cn (J.K.S.); gichira@wbgcas.cn (A.W.G.); cmulili90@gmail.com (C.M.K.); 2 University of Chinese Academy of Sciences, Beijing 100049, China; 3 Sino-African Joint Research Center, Chinese Academy of Sciences, Wuhan 430074, China

**Keywords:** Balsaminaceae, chloroplast genome, *Hydrocera triflora*, *Impatiens pinfanensis*, phylogenetic analyses

## Abstract

The family Balsaminaceae, which consists of the economically important genus *Impatiens* and the monotypic genus *Hydrocera*, lacks a reported or published complete chloroplast genome sequence. Therefore, chloroplast genome sequences of the two sister genera are significant to give insight into the phylogenetic position and understanding the evolution of the Balsaminaceae family among the Ericales. In this study, complete chloroplast (cp) genomes of *Impatiens pinfanensis* and *Hydrocera triflora* were characterized and assembled using a high-throughput sequencing method. The complete cp genomes were found to possess the typical quadripartite structure of land plants chloroplast genomes with double-stranded molecules of 154,189 bp (*Impatiens pinfanensis*) and 152,238 bp (*Hydrocera triflora*) in length. A total of 115 unique genes were identified in both genomes, of which 80 are protein-coding genes, 31 are distinct transfer RNA (tRNA) and four distinct ribosomal RNA (rRNA). Thirty codons, of which 29 had A/T ending codons, revealed relative synonymous codon usage values of >1, whereas those with G/C ending codons displayed values of <1. The simple sequence repeats comprise mostly the mononucleotide repeats A/T in all examined cp genomes. Phylogenetic analysis based on 51 common protein-coding genes indicated that the Balsaminaceae family formed a lineage with Ebenaceae together with all the other Ericales.

## 1. Introduction

The family Balsaminaceae of the order Ericales contains only two genera, *Impatiens* Linnaeus (1753:937) and *Hydrocera* Wight and Arnott (1834:140) and are predominantly perennial and annual herbs [1]. The monotypic genus *Hydrocera*, with a single species *Hydrocera triflora*, is characterized by actinomorphic flowers, a pentamerous calyx and corolla without any fusion between perianth parts, contrary to highly similar sister genus *Impatiens* whose flowers are highly zygomorphic [2]. *Impatiens*, one of the largest genera in angiosperms, consists of over 1000 species [3,4,5,6] primarily distributed in the Old World tropics, subtropics and temperate regions, but also in Europe, and central and North America [5,7]. In contrast, the sister *Hydrocera*, which is a semi-aquatic plant, is restricted to the lowlands of Indo-Malaysia [1]. Besides, the geographical regions, including south-east Asia, the eastern Himalayas, tropical Africa, Madagascar, southern India and Sri Lanka occupied by *Impatiens*, have been identified as diversity hotspots [7,8]. Recently, numerous new species have been recorded within these regions each year [9,10,11,12,13,14].

The controversial nature of classification of the genus *Impatiens* [1,15], for example different floral characters, its hybridization nature and species radiation, has made it under-studied. The species in prolific genus *Impatiens* are economically used as ornamentals, medicinal, as well as experimental research plant materials [16]. Additionally, previous studies have shown the genus *Impatiens* to possess potential anticancer compounds by decreasing patients’ cancer cell count and increasing their life span and body weight [17]. The glanduliferins A and B isolated from the stem act to inhibit the growth of human cancer cells for growth inhibitory activity of human cancer cells [18]. As well, some polyphenols from *Impatiens* stems have showed antioxidant and antimicrobial activities [19].

In angiosperms, the chloroplast genome (cp) typically has a quadripartite organization consisting of a small single copy (SSC, 16–27 kb) and one large single copy (LSC) of about 80–90 kb long separated by two identical copies of inverted repeats (IRs) of about 20–88 kb with the total complete chloroplast genome size ranging from 72 to 217 kb [20,21,22]. Most of the complete cp genomes contains 110–130 distinct genes, with approximately 80 genes coding for proteins, 30 tRNA and 4 rRNA genes [21]. In addition, due to the highly conserved gene order and gene content, they have been used in plant evolution and systematic studies [23], determining evolutionary patterns of the cp genomes [24], phylogenetic analysis [25,26], and comparisons of angiosperm, gymnosperm, and fern families [27]. Moreover, the cp genomes are useful in genetic engineering [28], phylogenetics and phylogeography of angiosperms [29], and estimation of the diversification pattern and ancestral state of the vegetation within the family [30].

The Ericales (Bercht and Presl) form a well-supported clade (Asterid) containing more than 20 families [31]. Up to now, complete cp genomes representing approximately half of the families in the order Ericales have been sequenced including: Actinidiaceae [32,33], Ericaceae [34,35], Ebenaceae [36], Sapotaceae [37], Primulaceae [38,39] Styracaceae [40], and Theaceae, Pentaphylacaceae, Sladeniaceae, Symplocaceae, Lecythidaceae [30]. In addition the *Impatiens* and *Hydrocera* intergeneric phylogenetic relationship has been done using chloroplast *atpB-rbcL* spacer sequences [4]. However, there are no reports of complete chloroplast genomes in the family Balsaminaceae to date. This limitation of genetic information has hindered the progress and understanding in taxonomy, phylogeny, evolution and genetic diversity of Balsaminaceae. Analyses of more cp genomes are needed to provide a robust picture of generic and familial relationships of families in order Ericales.

This study aims to determine the complete sequences of the chloroplast genomes of *I. pinfanensis* (Hook. f.) and *H. triflora* using a high-throughput sequencing method. Additionally, comparisons with other published cp genomes in the order Ericales will be made in order to determine phylogenetic relationships among the representatives of Ericales.

## 2. Results and Discussion

### 2.1. The I. pinfanensis and H. triflora Chloroplast Genome Structure and Gene Content

The complete chloroplast genomes of *I. pinfanensis* and *H. triflora* share the common feature of possessing a typical quadripartite structure composed of a pair of inverted repeats (IRs) separating a large single copy (LSC) and a small single copy (SSC), similar to other angiosperm cp genomes [23]. The cp genome size of *I. pinfanensis* is 154,189 bp, with a pair of inverted repeats (IRs) of 17,611 bp long that divide LSC of 83,117 bp long and SSC of 25,755 bp long (Table 1). On the other hand, the *H. triflora* complete cp genome is 152,238 bp in length comprising a LSC region of 84,865 bp in size, a SSC of 25,622 bp size, and a pair of IR region 18,082 bp each in size. The overall guanine-cytosine (GC) contents of *I. pinfanensis* and *H. triflora* genomes are 36.8% and 36.9% respectively. Meanwhile, the GC contents in the LSC, SSC, and IR regions are 34.5%/34.7%, 29.3%/29.9%, and 43.1%/43.1% respectively.

**Figure 1:**
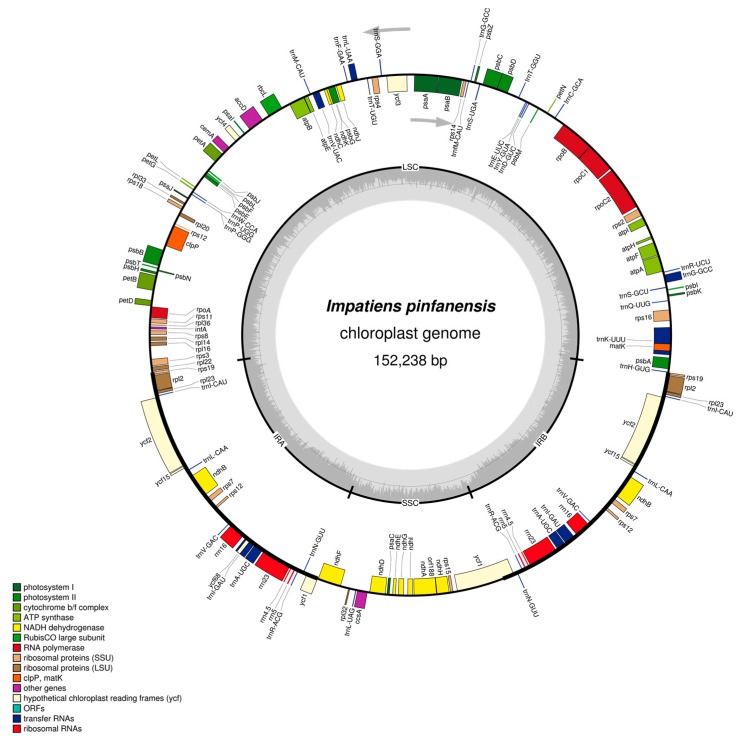
Gene map of the *Impatiens pinfanensis* chloroplast genome. Genes lying outside of the circle are transcribed clockwise, while genes inside the circle are transcribed counterclockwise. The colored bars indicate different functional groups. The dark gray area in the inner circle corresponds to GC content while the light gray corresponds to the adenine-thymine (AT) content of the genome.

**Figure 2:**
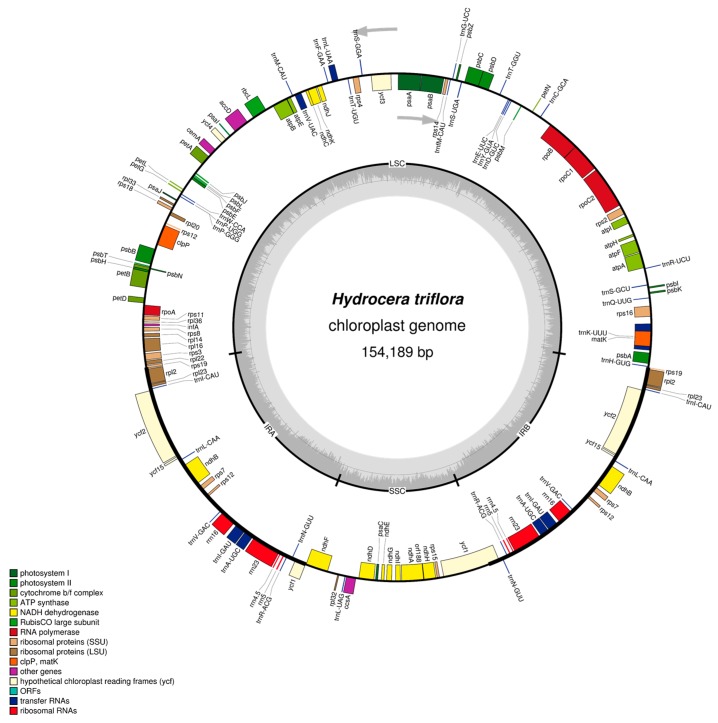
Gene map of the *Hydrocera triflora* chloroplast genome. Genes lying outside of the circle are transcribed clockwise, while genes inside the circle are transcribed counterclockwise. The colored bars indicate different functional groups. The dark gray area in the inner circle corresponds to (guanine cytosine) GC content while the light gray corresponds to the AT content of the genome.

Like in typical angiosperms, both *I. pinfanensis* and *H. triflora* cp genomes encode 115 total distinct genes of which 80 are protein coding, 31 distinct tRNA and four distinct rRNA genes. Of these 62 genes coding for proteins and 23 tRNA genes were located in the LSC region, seven protein-coding genes, all the four rRNA genes and seven tRNA genes were replicated in the IR regions, while the SSC region was occupied by 11 protein-coding genes and one tRNA gene. The *ycf1* gene was located at the IR and SSC boundary region (Figure 1 and Figure 2).

Among the 115 unique genes in *I. pinfanensis* and *H. triflora* cp genomes, 14 genes contain one intron, comprised of eight genes coding for proteins (*atpF*, *rpoC1*, *rpl2*, *petB*, *rps16*, *ndhA*, *ndhB*, *ndhK*) and six tRNAs (*trnL-UAA*, *trnV-UAC*, *trnK-UUU*, *trnI-GAU*, *trnG-GCC* and *trnA-UGC*) (Table 2), while *ycf3*, *clpP* and *rps12* genes each contain two introns. These genes have maintained intron content in other angiosperms. The trans-splicing gene *rps12* has its 5′exon located in LSC, whereas the 3′exon is located in the IRs, which is similar to that in *Diospyros* species (Ebenaceae) [36,41] and *Actinidia chinensis* (Actinidiaceae) [41]. Oddly, *rps19* and *ndhD* genes in both species begin with uncommon start codons GTG and ACG respectively, which is consistent with previous reports in other plants [36]. However, the standard start codon can be restored through RNA editing process [42,43].

The complete cp genome of *I. pinfanensis* and *H. triflora* were found to be similar, although some slight variations such as genome size, gene loss and IR expansion and contraction factors were detected, despite the two species being from the same family Balsaminaceae. For instance, *H. triflora* cp genome is 1951 bp smaller than that of sister species *I. pinfanensis*. The SSC region of *I. pinfanensis* is shorter (17,611 bp) compared to that of *H. triflora*, which is 18,082 bp long. The GC content of *H. triflora* is slightly higher (36.9%) than that of *I. pinfanensis* (36.8%). Both species possess highest GC values in the IR regions (43.1%) compared to LSC and SSC region showing the lowest values (34.5%/34.7% and 29.3%/29.9%) respectively. The IR region is more conserved than the single copy region (SSC) in both species, due to presence of conserved rRNA genes in the IR region, which is also the reason for its high GC content. Both cp genomes are AT-rich with the genome organization and content of the two species almost the same and highly conserved, these results are similar to those of other recently published Ericales chloroplast genomes [34,36].

### 2.2. Codon Usage

The relative synonymous codon usage (RSCU) has been divided into four models, i.e., RSCU value of less than 1.0 (lack of bias), RSCU value between 1.0 and 1.2 (low bias), RSCU value between 1.2 and 1.3 (moderately bias) and RSCU value greater than 1.3 (highly bias) [44,45]. To determine codon usage, we selected 52 shared protein-coding genes between *I. pinfanensis* and *H. triflora* with length of >300 bp for calculating the effective number of codons. As shown in (Table 3), the relative synonymous codon usage (RSCU) and codon usage revealed biased codon usage in both species with values of 30 codons showing preferences (<1) except tryptophan and methionine, with 29 having A/T ending codons. The TAA stop codon was found to be preferred. All the protein-coding genes contained 22,900 and 22,995 codons in *I. pinfanensis* and *H. triflora* cp genomes respectively. In addition, our results indicated that 2408 and 2439 codons encode leucine while 253 and 259 encode cysteine in *I. pinfanensis* and *H. triflora* cp genomes as the most and least frequently universal amino acids respectively. The Number of codons (Nc) of the individual PCGs varied from *petD* (37.10) to *ycf3* (54.84) and *rps18* (32.11) to *rpl2* (54.24) in *I. pinfanensis* and *H. triflora* respectively (Table S1). Like recently reported in cp genomes of higher plants, our study showed that there was bias in the usage of synonymous codons except tryptophan and methionine. Our result is in line with previous findings of codon usage preference for A/T ending in other land plants [46,47].

### 2.3. SSR Analysis Results

Analysis of SSR occurrence using the microsatellite identification tool (MISA) detected Mono-, di-, tri-, tetra-, penta- and hexa-nucleotides categories of SSRs in the cp genomes of eight Ericales. A total of 197 and 159 SSRs were found in the *I. pinfanensis* and *H. triflora* cp genomes respectively. Not all the SSR types were identified in all the species, Penta and hexanucleotide repeats were not found in *I. pinfanensis*, *Diospyros lotus*, and *Pouteria campechiana*, while only hexanucleotides were not identified in *Ardisia polysticta* and *Barringtonia fusicarpa* (Table 4). Among the SSR types discovered mononucleotide repeat units were highly represented, which were found 180 and 141 times in *I. pinfanensis* and *H. triflora* respectively. Most of the mononucleotide repeats consisting of A or T were most common (117–176 times), whereas C/G were less in number (1–8 times), and all the dinucleotide repeat sequences in all the species were AT repeats. This result is consistent with previous reports, which showed most angiosperm cp genome to be AT-rich [36,38,48].

### 2.4. Selection Pressure Analysis of Evolution

**Figure 3:**
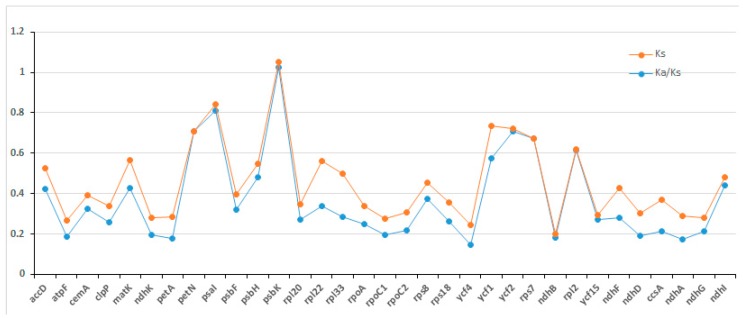
Non-synonymous (Ka) and synonymous (Ks) substitution rates and Ka/Ks ratio between *I. pinfanensis* and *H. triflora*. One gene *psbK* had Ka/Ks ratio greater than 1.0, whereas all the other genes were less than 1.0.

The ratio of Synonymous (Ks) and non-synonymous (Ka) Substitution can determine whether the selection pressure has acted on a particular protein-coding sequence. Eighty common protein-coding genes shared by *I. pinfanensis* and *H. triflora* genomes were used. As suggested by Makałowski and Boguski [49] the Ka/Ks values are less than one in protein-coding genes as a result of less frequent non-synonymous (Ka) nucleotide Substitutions than the Synonymous (Ks) substitutions (Table S2). We found that the Ka/Ks values of the two species were low (<1) approaching zero, except for one gene *psbK* found in the LSC region, which has a ratio of 1.0259 (Figure 3). This indicates a negative selection all genes except *psbK* gene and shows that the protein-coding genes in both species are quite highly conserved (Table S2). The LSC, SSC, and IR regions average Ks values between the two species were 0.0995, 0.0314, and 0.1334 respectively. Based on Ka/Ks comparison among the regions, only *ycf1* gene in IR region and most of the genes in the LSC and SSC regions revealed higher Ks values. The higher Ks values signaled that on average more genes found in the SSC region have experienced higher selection pressures in contrast to other cp genome regions (LSC and IR). The non-synonymous (Ka) value varied from 0.005 (*psbE*) to 0.0927 (*ycf1*) while Ks ranged from 0.058 (*psbN*) to 0.2944 (*ndhE*). Based on sequence similarity among the IR, SSC and LSC regions, the IR region was more conserved. This is in agreement with previous reports that found out that IR region diverged at a slower rate than the LSC and SSC regions as a result of frequent recombinant events taking place in IR region leading to selective constraints on sequence homogeneity [50,51].

### 2.5. IR Expansion and Contraction

**Figure 4:**
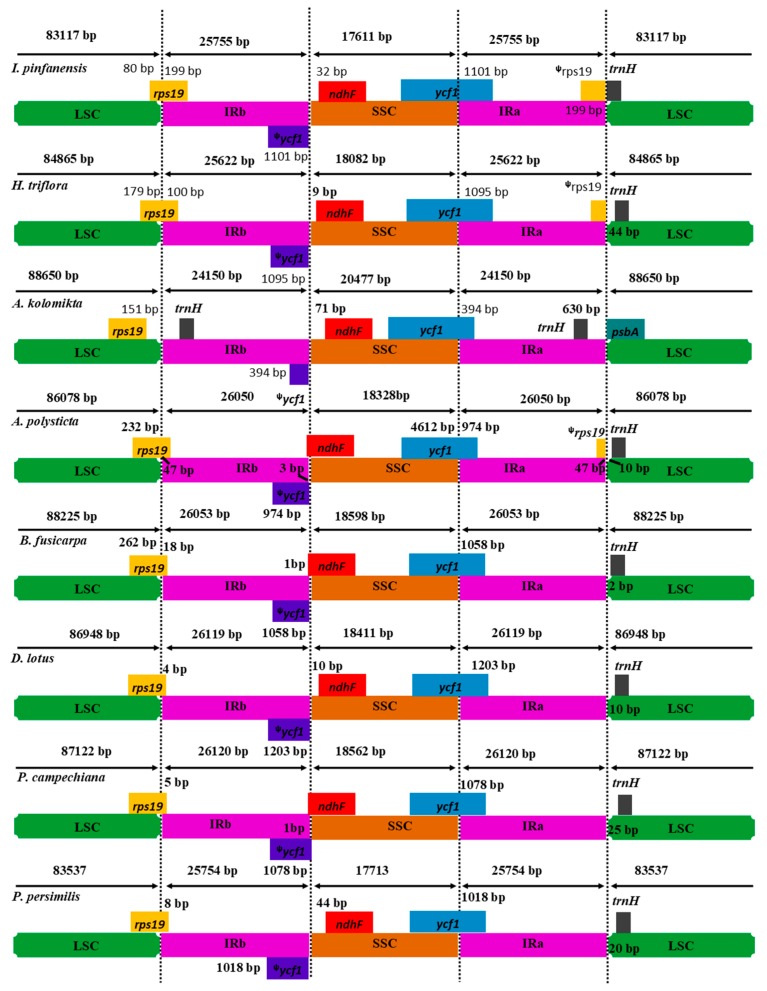
Comparison of IR, LSC and SSC border regions among eight Ericales cp genomes. The IRb/SSC junction extended into the *ycf1* genes creating various lengths of *ycf1* pseudogenes among the eight cp genomes. The numbers above, below or adjacent to genes shows the distance between the ends of genes and the boundary sites. The figure features are not to scale. ᵠ indicates a pseudogene.

Despite of the highly conserved nature of the angiosperms inverted repeat (IRa/b) regions, the contraction or expansion at the IR junction are the usual evolutionary events resulting in varying cp genome sizes [52,53]. In our study, the IR/SSC and IR/LSC borders of *I. pinfanensis* and *H. triflora* were compared to those of the other six Ericales representatives (*P. persimilis*, *P. campechiana*, *D. lotus*, *B. fusicarpa*, *A. kolomikta* and *A. polysticta*) to identify the IR expansion or contraction (Figure 4). The IRb/SSC boundary expansions in all the eight species extended into the *ycf1* genes creating long ᵠ*ycf1* pseudogene fragments with varying length. The *ycf1* pseudogene length in *I. pinfanensis* is 1101 bp, 1095 bp in *H. triflora*, 394 bp in *A. kolomikta*, 974 bp in *A. polysticta*, 1058 bp in *B. fusicarpa*, 1203 bp in *D. lotus*, 1078 bp in *P. campechiana* and 1018 bp in *P. persimilis*. Additionally, the *ndhF* gene is situated in the SSC region in *I. pinfanensis*, *H. triflora*, *A. kolomikta*, *D. lotus*, and *P. persimilis*, and it ranges from 32 bp, 9 bp, 71 bp, 10 bp and 44 bp away from the IRb/SSC boundary region respectively, but this gene formed an overlap with the *ycf1* pseudogene in *A. polystica*, *B. fusicarpa* and *P. campechiana* cp genomes sharing some nucleotides of 3 bp, 1 bp and 1 bp in that order. The *rps19* gene is located at the /IRb/LSC junction, of *I. pinfanensis*, *H. triflora* and of the other five cp genomes, apart from *A. kolomikta* in which this gene is found in the LSC region, 151 bp gap from the LSC/IRb junction. Moreover, the occurrence of *rps19* gene at the LSC/IRb junction resulted in partial duplication of this gene at the corresponding region (IRa/LSC border) in *I. pinfanensis*, *H. triflora*, and *A. polysticta* cp genomes. The *trnH* gene is detected in the LSC region in *I. pinfanensis* and *H. triflora*. However, complete gene rearrangement of this *trnH* gene was observed resulting in complete duplication in the IR in the *A. kolomikta* chloroplast genome, 630 bp apart from the IR/LSC junction with *psbA* gene extending towards LSC/IRa border, however this gene is found in the LSC regions of the other five chloroplast genomes.

The border regions of the Ericales revealed that the *I. pinfanensis* and *H. triflora* cp genomes varied a little compared to other analyzed cp genomes. As shown in Figure 4, our analyses confirmed the IR evolution as revealed by the incomplete *rps19* gene, which was duplicated in the IR region in *I. pinfanensis*, *H. triflora*, and *A. polysticta*. Conversely, this *rps19* gene was not duplicated among the remaining representatives of Ericales cp genomes. In a recent study [36,54] found that the *trnH* gene duplication occurs in Actinidiaceae, and Ericaceae. This duplication of genes in the LSC/IRb junction and the IRa/LSC junction would be of great importance in systematic studies. Furthermore, the *rps19* gene at the LSC/IRb in *I. pinfanensis* and *H. triflora* is largely extended into the IRb region (199 bp and 100 bp) respectively. The SSC region of *I. pinfanensis* is 471 bp smaller than that of sister species *H. triflora*, but also smallest among the other species used in this study. Additionally, the *I. pinfanensis* LSC region is smaller than that of other species. Previous studies have shown that there is expansion of single copy (SC) and IR regions of angiosperms cp genomes during evolution [50,55], the *I. pinfanensis* and *H. triflora* cp genomes revealed that the border areas were highly conserved despite of slight genome size differences between the two species.

### 2.6. Phylogenetic Analysis

**Figure 5:**
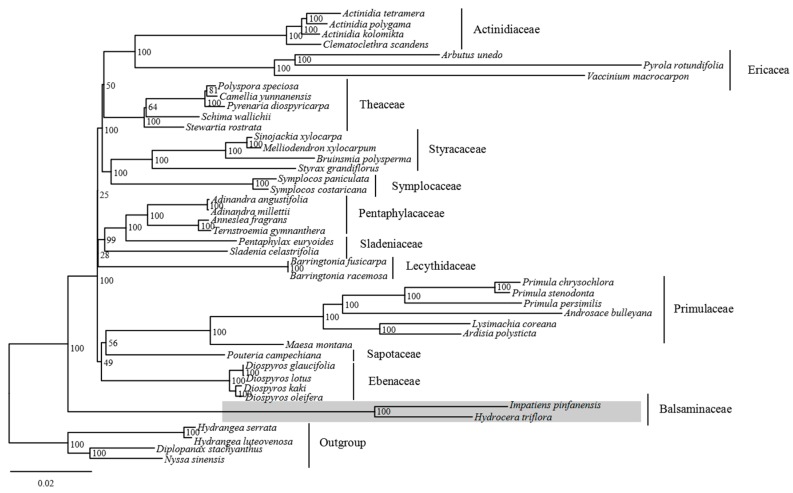
Phylogenetic relationships based on 51 common protein-coding genes of 38 representative species from order Ericales and four Cornales as Outgroup species with maximum likelihood. The numbers associated with the nodes indicate bootstrap values tested with 1000 replicates.

Phylogenetic relationships within the order Ericales have been resolved in recent published reports but the position of Balsaminaceae still remains controversial [33,35,36,37,38,39,40]. In our study, the phylogenetic relationship of *I. pinfanensis*, and *H. triflora* and 38 other species of Ericales downloaded from GenBank (Table S3) was determined, with four cp genomes sequences belonging to Cornales being used as Outgroup species. Fifty-one common protein-coding sequences in all the selected cp genomes employed a single alignment data matrix of a total 35,548 characters (Supplementary Materials File S4). Almost all the nodes in the phylogenetic tree showed a strong bootstrap support. Though, Sapotaceae and Ebenaceae had low support (bootstrap < 70), this could be as a result of fewer samples in these families (Figure 5). *I. pinfanensis* and *H. triflora* as sister taxa (Balsaminaceae) formed the basal family of Ericales with intensive support. In general, all the 38 species together with the two Balsaminaceae family species formed a lineage (Ericales) recognizably discrete from the four outgroup species (Cornales). All the species grouped together into 10 clades corresponding to the 10 families in order Ericales according to APGIV system [31]. This study will provide resources for species identification and resolution of deeper phylogenetic branches among *Impatiens* and *Hydrocera* genera.

## 3. Materials and Methods

### 3.1. Plant Materials and DNA Extraction

Total genomic DNA was extracted from fresh leaves of the *I. pinfanensis* and *H. triflora* collected from Hubei province (108°42′19′′ E, 30°12′33′′ N) and Hainan province (110°18′57′′ E, 19°23′10′′ N) in China using a modified cetyltrimethylammonium bromide (CTAB) method [56]. The DNA quality was checked using spectrophotometry and their integrity examined by electrophoresis in 2% agarose gel. The voucher specimens (HIB-lzz07, HIB-lzz18) were deposited at the Wuhan Botanical Garden herbarium (HIB).

### 3.2. Chloroplast Genome Sequence Assembly and Annotation

The pair-end libraries were constructed using the Illumina Hiseq 2500 platform at NOVOgene Company (Beijing, China) with an average insert size of approximately 150 bp for each genome. The high-quality reads were filtered from Illumina raw reads using the PRINSEQ lite v0.20.4 (San Diego State University, San Diego, CA, USA) [57] (phredQ ≥ 20, Length ≥ 50), then assembled with closely related species cp genome using a BLASTn (with *E* value of 10^−6^) with *Primula chrysochlora* (NC_034678) and *Diospyros lotus* (NC_030786) as reference species. In addition, the software Velvet v1.2.10 (Wellcome Trust Genome Campus, Hinxton, Cambridge, UK) [58] was used to assemble the obtained reads with K-mer length of 99–119. Then, consensus sequences with reference chloroplast genome was mapped using GENEIOUS 8.0.2 (Biomatters Ltd., Auckland, New Zealand) [59]. We used the online software local blast to verify the single copy (SC) and inverted repeat (IR) boundary regions of the assembled sequences.

The annotations of the complete cp genomes were performed using DOGMA (Dual Organellar GenoMe Annotator, University of Texas at Austin, Austin, TX, USA) [60]. The start and stop codons positions were further checked by local blast searches. Further, the tRNAs locations were confirmed with tRNAscan-SE v1.23 (http://lowelab.ucsc.edu/tRNAscan-SE/) [61]. The circular cp genome maps were generated using an online program (OGDrawV1.2, Max planck Institute of Molecular Plant Physiology, Potsdam, Germany) OrganellarGenomeDraw [62] with default settings plus manual corrections. Putative tRNAs, rRNAs and protein-coding genes were corrected by comparing them with the more similar reference species *Primula chrysochlora* (NC_034678) and *Diospyros lotus* (NC_030786) resulting from BLASTN and BLASTX searches against the nucleotide database NCBI (https://blast.ncbi.nlm.nih.gov/). The cp genome sequences were submitted to GenBank database, accession numbers *I. pinfanensis* (MG162586) and *H. triflora* (MG162585).

### 3.3. Genome Comparison and Structure Analyses

The IR and SC boundary regions of *I. pinfanensis* and *H. triflora*, and the other six Ericales species were compared and examined. For synonymous codon usage analysis, about 52 protein-coding genes of length > 300 bp were chosen. Online program CodonW1.4.2 (http://downloads.fyxm.net/CodonW-76666.html) was used to investigate the Nc and RSCU parameters. The simple sequence repeats (SSRs) of the two study species and other Ericales representatives were detected using MISA software [63] with SSR search parameters set same as Gichira et al. [48].

### 3.4. Substitution Rate Analysis—Synonymous (Ks) and Non-Synonymous (Ka)

We examined substitution rates synonymous (Ks) and non-synonymous (Ka) using Model Averaging in the KaKs_Cal-culator program (Institute of Genomics, Chinese Academy of Sciences, Beijing, China) [64]. Eighty common protein-coding genes shared by the *I. pinfanensis* and *H. triflora* were aligned separately using Geneious software v5.6.4 (Biomatters Ltd., Auckland, New Zealand) [59].

### 3.5. Phylogenetic Analyses

To locate the phylogenetic positions of *I. pinfanensis* and *H. triflora* (Balsaminaceae) within order Ericales, the chloroplast genome sequences of 38 species belonging to order Ericales and four Cornales species as outgroups, were used to reconstruct a phylogenetic relationships tree. The Phylogenetic tree was performed based on maximum likelihood (ML) analysis using RAxMLversion 8.0.20 (Scientific Computing Group, Heidelberg Institute for Theoretical Studies, Institute of Theoretical Informatics, Karlsruhe Institute of Technology, Karlsruhe, Germany) [65]. Consequently, based on the Akaike information criterion (AIC), the best-fitting substitution models (GTR + I + G) were selected (p-inv = 0.47, and gamma shape = 0.93) from jModelTest v2.1.7 [66]. The bootstrap test was performed in algorithm of RAxML with 1000 replicates.

## 4. Conclusions

The cp genomes of *I. pinfanensis*, and *H. triflora* from the family Balsaminaceae provide novel genome sequences and will be of benefit as a reference for further complete chloroplast genome sequencing within the family. The genome organization and gene content are well conserved typical of most angiosperms. Fifty protein-coding sequences, shared by selected species from Ericales as well as our study species, were used to construct the phylogenetic tree using the maximum likelihood (ML). Majority of the nodes showed strong bootstrap support values, and the few nodes with low support, should be solved using other methods (e.g., restriction-site-associated DNA sequencing). The two species (*I. pinfanensis*, and *H. triflora*) were placed close to each other. These findings strongly support Balsaminaceae as a basal family of the order Ericales. Lastly, the Balsaminaceae (*I. pinfanensis*, and *H. triflora*) has a relationship with the other 38 species, which are all grouped into one Clade (Ericales). This study will be of value in determining genome evolution and understanding phylogenomic relationships within Ericales and give precious resources for the evolutionary study of Balsaminaceae.

## Figures and Tables

**Table 1:** Comparison of the chloroplast genomes of *Impatiens pinfanensis* and *Hydrocera triflora*.

Species	*Impatiens pinfanensis*	*Hydrocera triflora*
Total Genome length (bp)	154,189	152,238
Overall G/C content (%)	36.8	36.9
Large single copy region	83,117	84,865
GC content (%)	34.5	34.7
Short single copy region	25,755	25,622
GC content (%)	29.3	29.9
Inverted repeat region	17,611	18,082
GC content (%)	43.1	43.1
Protein-Coding Genes	80	80
tRNAs	31	31
rRNAs	4	4
Genes with introns	17	17
Genes duplicated by IR	18	18

**Table 2:** Genes encoded in the *Impatiens pinfanensis* and *Hydrocera triflora* Chloroplast genomes.

Group of Genes	Gene Name
rRNA genes	*rrn16*(×2), *rrn23*(×2), *rrn4.5*(×2), *rrn5*(×2),
tRNA genes	*trnA-UGC* * (×2), *trnC-GCA*, *trnD-GUC*, *trnE-UUC*, *trnF-GAA*, trnG-GCC *, *trnG-UCC*, *trnH-GUG*, *trnI-CAU*(×2), *trnI-GAU* * (×2), *trnK-UUU* *, *trnL-CAA*(×2), *trnL-UAA* *, *trnL-UAG*, *trnfM-CAU*, *trnM-CAU*, *trnN-GUU*(×2), *trnP-GGG trnP-UGG*, *trnQ-UUG*, *trnR-ACG*(×2), *trnR-UCU*, *trnS-GCU*, *trnS-GGA*, *trnS-UGA*, *trnT-GGU*, *trnT-UGU*, *trnV-GAC*(×2), *trnV-UAC* *, *trnW-CCA*, *trnY-GUA*
Ribosomal small subunit	*rps2*, *rps3*, *rps4*, *rps7*(×2), *rps8*, *rps11*, *rps12*_*5’end*, *rps12*_*3’end* * (×2), *rps14*, *rps15*, *rps16* *, *rps18*, *rps19*
Ribosomal large subunit	*rpl2* * (×2), *rpl14*, *rpl16*, *rpl20*, *rpl22*, *rpl23*(×2), *rpl32*, *rpl33*, *rpl36*
DNA-dependent RNA polymerase	*rpoA*, *rpoB*, *rpoC1* *, *rpoC2*
Large subunit of rubisco	*rbcL*
Photosystem I	*psaA*, *psaB*, *psaC*, *psaI*, *psaJ*, *ycf3* **
Photosystem II	*psbA*, *psbB*, *psbC*, *psbD*, *psbE*, *psbF*, *psbH*, *psbI*, *psbJ*, *psbK*, *psbL*, *psbM*, *psbN*, *psbT*, *psbZ*
NADH dehydrogenase	*ndhA* *, *ndhB* * (×2), *ndhC*, *ndhD*, *ndhE*, *ndhF*, *ndhG*, *ndhH*, *ndhI*, *ndhJ*, *ndhK*
Cytochrome b/f complex	*petA*, *petB* *, *petD*, *petG*, *petL*, *petN*
ATP synthase	*atpA*, *atpB*, *atpE*, *atpF* *, *atpH*, *atpI*
Maturase	*matK*
Subunit of acetyl-CoA carboxylase	*accD*
Envelope membrane protein	*cemA*
Protease	*clpP* **
Translational initiation factor	*infA*
c-type cytochrome synthesis	*ccsA*
Conserved open reading frames (*ycf*)	*ycf1*, *ycf2*(×2), *ycf4*, *ycf15*(×2)

Genes with one or two introns are indicated by one (*) or two asterisks (**), respectively. Genes in the IR regions are followed by the (×2) symbol.

**Table 3:** Codon usage in *Impatiens pinfanensis* and *Hydrocera triflora* chloroplast genomes.

Amino Acid	Codon	Number		RSCU		Amino Acid	Codon	Number		RSCU	
		*I. pinfanensis*	*H. triflora*	*I. pinfanensis*	*H. triflora*			*I. pinfanensis*	*H. triflora*	*I. pinfanensis*	*H. triflora*
Phe	UUU	913	908	**1.40**	**1.38**	Ser	UCU	482	482	**1.69**	**1.67**
	UUC	387	406	0.60	0.62		UCC	252	264	0.88	0.92
Leu	UUA	854	842	**2.11**	**2.07**		UCA	360	324	**1.26**	**1.12**
	UUG	468	486	**1.16**	**1.20**		UCG	142	181	0.50	0.63
	CUU	517	503	**1.28**	**1.24**	Pro	CCU	376	371	**1.59**	**1.58**
	CUC	160	162	0.40	0.40		CCC	175	167	0.74	0.71
	CUA	310	315	0.77	0.78		CCA	294	290	**1.24**	**1.23**
	CUG	121	128	0.30	0.32		CCG	103	112	0.43	0.48
Ile	AUU	1035	1020	**1.54**	**1.52**	Thr	ACU	493	500	**1.70**	**1.74**
	AUC	359	376	0.53	0.56		ACC	198	180	0.68	0.63
	AUA	624	611	0.93	0.91		ACA	358	368	**1.24**	**1.28**
Met	AUG	547	548	1.00	1.00		ACG	108	104	0.37	0.36
Val	GUU	482	469	**1.55**	**1.52**	Ala	GCU	580	593	**1.86**	**1.85**
	GUC	134	135	0.43	0.44		GCC	183	191	0.59	0.60
	GUA	457	457	**1.47**	**1.48**		GCA	346	353	**1.11**	**1.10**
	GUG	167	174	0.54	0.56		GCG	141	143	0.45	0.45
Tyr	UAU	704	697	**1.64**	**1.65**	Cys	UGU	191	196	**1.53**	**1.51**
	UAC	155	146	0.36	0.35		UGC	58	63	0.47	0.49
TER	UAA	41	44	**1.50**	**1.63**	TER	UGA	18	18	0.66	0.67
	UAG	23	19	0.84	0.70	Trp	UGG	412	412	1.00	1.00
His	CAU	405	421	**1.54**	**1.57**	Arg	AGA	406	407	**1.81**	**1.77**
	CAC	121	114	0.46	0.43		AGG	134	143	0.60	0.62
Gln	CAA	627	626	**1.54**	**1.53**	Arg	CGU	302	299	**1.35**	**1.30**
	CAG	186	192	0.46	0.47		CGC	88	95	0.39	0.41
Asn	AAU	885	868	**1.59**	**1.57**		CGA	317	333	**1.41**	**1.45**
	AAC	231	238	0.41	0.43		CGG	98	103	0.44	0.45
Lys	AAA	976	978	**1.55**	**1.54**	Ser	AGU	363	72	**1.27**	**1.29**
	AAG	284	289	0.45	0.46		AGC	110	108	0.39	0.37
Asp	GAU	720	737	**1.64**	**1.64**	Gly	GGU	525	525	**1.33**	**1.35**
	GAC	159	160	0.36	0.36		GGC	160	165	0.40	0.42
Glu	GAA	914	929	**1.55**	**1.55**		GGA	639	625	**1.62**	**1.61**
	GAG	264	272	0.45	0.45		GGG	258	238	0.65	0.61

RSCU: Relative synonymous Codon Usage. RSCU > 1 are highlighted in bold.

**Table 4:** SSR types and amount in the *Impatiens pinfanensis* and *Hydrocera triflora* Chloroplast genomes.

SSR Type	Repeat Unit	Amount							
		*Impatiens pinfanensis*	*Hydrocera triflora*	*Actinidia kolomikta*	*Ardisia polysticta*	*Diospyros lotus*	*Barringtonia fusicarpa*	*Pouteria campechiana*	*Primula persimilis*
Mono	A/T	176	139	117	153	146	154	161	134
	C/G	4	2	4	4	4	8	1	4
Di	AT/AT	8	9	8	5	3	13	11	6
Tri	AAG/CTT	1	0	0	0	0	0	1	1
	AAT/ATT	3	3	2	1	1	2	4	0
	AGC/CTG	0	0	0	0	1	0	0	0
Tetra	AAAG/CTTT	1	0	3	2	1	3	1	1
	AAAT/ATTT	2	3	3	3	4	3	6	2
	AATG/ATTC	1	0	0	1	0	0	0	0
	AATT/AATT	1	0	1	0	0	0	1	0
	AGAT/ATCT	1	0	0	0	0	0	0	0
	AAGT/ACTT	0	1	0	0	0	1	0	0
	AACT/AGTT	0	0	0	1	0	0	0	0
	AATC/ATTG	0	0	2	0	1	1	0	0
	AAAC/GTTT	0	0	0	0	0	0	1	0
	AAGG/CCTT	0	0	0	0	0	0	1	0
Penta	AATAC/ATTGT	0	1	0	0	0	0	0	0
	AAAAT/ATTTT	0	0	1	0	0	0	0	0
	AAATT/AATTT	0	0	0	1	0	0	0	0
	AATGT/ACATT	0	0	0	0	0	1	0	0
	AATAT/ATATT	0	0	0	0	0	0	0	1
Hexa	AATCCC/ATTGGG	0	1	0	0	0	0	0	0
	AGATAT/ATATCT	0	0	0	0	0	0	0	1
	AAGATG/ATCTTC	0	0	1	0	0	0	0	0
Total		197	159	143	171	161	187	188	150

## Supplementary Materials

Supplementary materials can be found at http://www.mdpi.com/1422-0067/19/1/319/s1.

## Abbreviations

IR	Inverted repeat
LSC	Large single copy
SSC	Small single copy
SSR	Simple sequence repeats
RSCU	Relative synonymous codon usage
