# Supplementary material for: Comparative Genomics of the Balsaminaceae Sister Genera Hydrocera triflora and Impatiens pinfanensis
Source: Int J Mol Sci. 2018 Jan 23;19(1):319. doi: 10.3390/ijms19010319 (PMC5796262; doi:10.3390/ijms19010319)
Supplement: Supplementary file 1 [file ijms-19-00319-s001.zip › ijms-260121-supplementary/ijms- 260121-supplmentary/ijms-260121-supplementary1-3.pdf]

International Journal of *Molecular Sciences*

MDPI

Table S1. The calculated parameter values for PCG in I. pinfanensis

Title	T3s	C3s	A3s	G3s	CAI	CBI	Fop	Nc	GC3s	GC	L_sym	L_aa	Gravy	Aromo
psbA	0.5263	0.2336	0.3175	0.0905	0.296	0.195	0.532	41.91	0.275	0.418	331	353	0.34136	0.135977
matK	0.4757	0.1514	0.4551	0.1854	0.161	-0.113	0.345	48.39	0.255	0.333	220	232	-0.19009	0.146552
atpA	0.4937	0.1385	0.4219	0.1684	0.192	-0.058	0.383	47.04	0.24	0.396	496	507	-0.06312	0.059172
atpF	0.4771	0.1569	0.4694	0.1908	0.15	-0.167	0.325	47.01	0.257	0.36	191	198	-0.3303	0.085859
atpI	0.4906	0.1651	0.4115	0.1198	0.156	-0.09	0.34	40.55	0.231	0.372	238	247	0.72834	0.121457
rps2	0.5077	0.1128	0.4509	0.1533	0.161	-0.164	0.32	43.82	0.203	0.371	222	236	-0.23559	0.072034
rpoC2	0.4695	0.1415	0.4603	0.1865	0.14	-0.186	0.304	47.58	0.245	0.361	1349	1385	-0.22563	0.083032
rpoC1	0.4762	0.1499	0.4615	0.1762	0.147	-0.143	0.323	49.7	0.246	0.383	684	707	-0.28458	0.09901
rpoB	0.4638	0.1133	0.4613	0.2219	0.147	-0.141	0.329	48.11	0.25	0.385	1037	1070	-0.25346	0.08785
psbD	0.5151	0.194	0.351	0.1422	0.254	0.068	0.456	45.99	0.275	0.425	331	353	0.364873	0.169972
psbC	0.4716	0.1778	0.3864	0.1498	0.187	-0.037	0.388	44.78	0.27	0.434	448	473	0.245877	0.143763
rps14	0.4026	0.1299	0.4375	0.2667	0.142	-0.054	0.385	45.57	0.313	0.423	96	100	-1.033	0.06
psaB	0.5104	0.1579	0.3691	0.1749	0.183	-0.114	0.351	47.52	0.261	0.407	689	734	0.128883	0.148501
psaA	0.4829	0.1801	0.3978	0.1375	0.201	-0.077	0.371	47.52	0.257	0.418	707	750	0.241733	0.132
ycf3	0.4925	0.1493	0.4701	0.198	0.142	-0.229	0.304	54.84	0.248	0.377	161	168	-0.48571	0.142857
rps4	0.4843	0.1321	0.4688	0.1655	0.152	-0.025	0.386	49.12	0.228	0.371	197	201	-0.64925	0.069652
ndhJ	0.5	0.1587	0.4091	0.2165	0.162	-0.203	0.289	51.34	0.275	0.397	149	158	-0.26076	0.14557
ndhK	0.5365	0.1245	0.4286	0.1489	0.171	-0.177	0.316	46.42	0.21	0.363	272	283	-0.24311	0.102474
ndhC	0.55	0.11	0.3953	0.1507	0.195	-0.052	0.36	43.92	0.198	0.35	111	120	1.068333	0.216667
atpE	0.5093	0.1389	0.4259	0.1383	0.17	-0.068	0.364	46.22	0.217	0.383	129	135	-0.09407	0.022222
atpB	0.4619	0.1597	0.4163	0.164	0.204	0.004	0.414	47.24	0.261	0.42	483	498	-0.00602	0.062249
rbcL	0.4961	0.1877	0.3775	0.1747	0.273	0.074	0.465	48.4	0.286	0.443	458	475	-0.28442	0.098947
accD	0.5812	0.1548	0.354	0.2112	0.179	-0.205	0.314	47.24	0.264	0.349	474	496	-0.50887	0.106855
ycf4	0.5099	0.1589	0.4275	0.1293	0.157	-0.083	0.36	44.44	0.223	0.368	175	184	0.233152	0.163043
cemA	0.5333	0.1611	0.4671	0.1508	0.18	-0.142	0.33	48.76	0.223	0.304	215	229	0.162446	0.152838

petA	0.5391	0.125	0.3891	0.1947	0.182	-0.096	0.35	46.57	0.242	0.382	314	320	-0.0825	0.078125
rps18	0.5063	0.0759	0.4881	0.16	0.098	-0.241	0.263	41.82	0.182	0.34	99	101	-0.82574	0.069307
rpl20	0.4554	0.1089	0.4457	0.2078	0.092	-0.206	0.281	47.43	0.237	0.368	114	117	-0.56239	0.076923
clpP	0.4581	0.1935	0.4497	0.1119	0.197	-0.076	0.361	52.87	0.246	0.412	183	194	0.137113	0.092784
psbB	0.5069	0.1544	0.379	0.1536	0.193	-0.066	0.378	46.49	0.249	0.434	481	508	0.135827	0.145669
petB	0.5191	0.1694	0.3598	0.12	0.224	-0.002	0.399	40.23	0.241	0.397	203	219	0.526941	0.141553
petD	0.4803	0.1053	0.4928	0.1048	0.174	-0.125	0.306	37.1	0.171	0.365	170	178	0.496629	0.11236
rpoA	0.4764	0.1527	0.5117	0.1727	0.152	-0.141	0.327	49.46	0.234	0.323	342	352	-0.26477	0.085227
rps11	0.4793	0.0909	0.4435	0.1238	0.157	-0.104	0.353	48.04	0.18	0.437	133	138	-0.41884	0.036232
rps8	0.4018	0.1964	0.4717	0.1379	0.111	-0.017	0.395	42.45	0.264	0.366	129	134	-0.40299	0.059701
rpl14	0.49	0.15	0.4227	0.1566	0.183	-0.016	0.39	49.22	0.237	0.399	118	122	-0.0582	0.040984
rpl16	0.4316	0.1158	0.52	0.1059	0.141	-0.023	0.407	42.21	0.177	0.417	113	120	-0.22333	0.083333
rps3	0.3865	0.2209	0.5774	0.0956	0.154	-0.064	0.383	47.95	0.234	0.349	209	218	-0.38395	0.091743
rpl22	0.4757	0.1165	0.5392	0.1163	0.143	-0.184	0.31	41.52	0.175	0.328	126	129	-0.34264	0.077519
rpl2	0.3906	0.2017	0.4163	0.195	0.14	-0.097	0.357	53.19	0.32	0.44	269	275	-0.61927	0.047273
ycf2	0.435	0.2164	0.3977	0.2599	0.159	-0.123	0.346	53.02	0.349	0.381	2172	2265	-0.4494	0.122737
ndhB	0.4286	0.2177	0.4316	0.121	0.161	-0.091	0.351	47.97	0.277	0.373	484	510	0.677255	0.129412
rps7	0.425	0.1333	0.5194	0.1282	0.186	-0.065	0.383	45.95	0.208	0.406	149	155	-0.54516	0.045161
ndhF	0.5433	0.1181	0.4369	0.129	0.152	-0.17	0.303	44.34	0.188	0.314	702	739	0.5682	0.161028
ccsA	0.5131	0.1011	0.5092	0.116	0.136	-0.261	0.257	43.72	0.162	0.3	296	312	0.588141	0.166667
ndhD	0.4755	0.1538	0.4731	0.0952	0.138	-0.153	0.312	44	0.202	0.335	465	500	0.7392	0.164
ndhE	0.5455	0.1477	0.4342	0.0469	0.164	-0.172	0.299	40.44	0.165	0.31	97	101	0.788119	0.089109
ndhG	0.5062	0.1125	0.4308	0.1442	0.128	-0.263	0.235	47.55	0.194	0.326	170	176	1.090341	0.136364
ndhI	0.5	0.1691	0.4602	0.1563	0.194	-0.092	0.361	47.25	0.241	0.354	158	165	-0.0497	0.115152
ndhA	0.4662	0.0997	0.5	0.1098	0.116	-0.176	0.28	41.73	0.167	0.33	347	361	0.755125	0.132964
ndhH	0.4886	0.1205	0.5139	0.1341	0.148	-0.14	0.323	46.21	0.19	0.369	368	393	-0.13868	0.114504
ycf1	0.4882	0.159	0.5652	0.149	0.152	-0.133	0.343	45.88	0.215	0.277	1779	1840	-0.55114	0.127717

Table S2. The calculated parameter values for each PCG in *H. triflora*

Title	T3s	C3s	A3s	G3s	CAI	CBI	Fop	Nc	GC3s	GC	L_sym	L_aa	Gravy	Aromo
psbA	0.5296	0.2401	0.3135	0.0811	0.302	0.211	0.541	41.52	0.275	0.418	331	353	0.334278	0.135977
matK	0.5062	0.1414	0.5014	0.164	0.161	-0.144	0.327	46	0.222	0.311	486	503	-0.14573	0.15507
atpA	0.4962	0.1392	0.4219	0.1684	0.198	-0.044	0.391	48.15	0.24	0.398	496	507	-0.08935	0.059172
atpF	0.4516	0.1548	0.46	0.2222	0.146	-0.168	0.321	41.07	0.28	0.367	193	198	-0.30556	0.070707
atpI	0.5024	0.1517	0.4301	0.1012	0.158	-0.084	0.345	42.29	0.206	0.364	238	247	0.705263	0.121457
rps2	0.5206	0.1082	0.4335	0.1645	0.181	-0.1	0.356	45.58	0.207	0.377	222	236	-0.22119	0.072034
rpoC2	0.4541	0.152	0.4494	0.1998	0.139	-0.176	0.31	49.16	0.265	0.37	1353	1386	-0.23961	0.078644
rpoC1	0.483	0.1485	0.4575	0.1816	0.15	-0.143	0.324	49.19	0.247	0.385	673	696	-0.32974	0.097701
rpoB	0.4637	0.1124	0.4554	0.2252	0.144	-0.153	0.321	48.24	0.253	0.388	1036	1070	-0.27907	0.082243
psbD	0.51	0.2067	0.332	0.1522	0.246	0.063	0.453	46.72	0.293	0.432	331	353	0.365722	0.169972
psbC	0.4691	0.1827	0.3842	0.1459	0.187	-0.024	0.395	44.53	0.272	0.435	448	473	0.257928	0.139535
rps14	0.4079	0.1184	0.4444	0.2632	0.14	-0.075	0.375	44.46	0.302	0.42	96	100	-0.995	0.06
psaB	0.5152	0.1531	0.3601	0.1861	0.185	-0.107	0.356	47.74	0.264	0.41	689	734	0.130518	0.149864
psaA	0.4736	0.1817	0.3915	0.1538	0.205	-0.075	0.372	48.44	0.27	0.424	707	750	0.251733	0.132
ycf3	0.4853	0.1544	0.4701	0.198	0.146	-0.208	0.315	53.38	0.253	0.381	162	169	-0.44497	0.142012
rps4	0.4904	0.1401	0.4688	0.1586	0.164	0.008	0.406	48.22	0.228	0.37	197	201	-0.62836	0.074627
ndhJ	0.5079	0.1587	0.422	0.1979	0.161	-0.207	0.289	46.69	0.262	0.388	149	158	-0.29684	0.14557
ndhK	0.5193	0.1288	0.4375	0.1568	0.155	-0.21	0.295	47.03	0.218	0.365	271	282	-0.19504	0.102837
ndhC	0.52	0.15	0.407	0.1233	0.202	0.007	0.396	45.47	0.216	0.353	111	120	1.0475	0.216667
atpE	0.5	0.1509	0.3925	0.1739	0.16	-0.099	0.346	46.88	0.252	0.388	127	133	0.013534	0.022556
atpB	0.4545	0.1671	0.4113	0.1689	0.205	-0.002	0.41	47.6	0.271	0.424	483	498	-0.01847	0.060241
rbcL	0.5168	0.1731	0.3881	0.1566	0.272	0.057	0.456	47.26	0.261	0.436	456	475	-0.288	0.098947
accD	0.5954	0.1603	0.349	0.2033	0.197	-0.182	0.329	48.07	0.26	0.341	477	498	-0.45763	0.110442
ycf4	0.4967	0.1569	0.4214	0.1441	0.156	-0.08	0.358	44.95	0.233	0.373	176	184	0.251087	0.163043
cemA	0.5193	0.1713	0.4459	0.1628	0.175	-0.147	0.324	44.45	0.241	0.311	216	229	0.233188	0.152838
petA	0.5315	0.1457	0.3789	0.1921	0.203	-0.037	0.387	46.36	0.259	0.394	313	320	-0.10344	0.08125
**rps18**	0.5185	0.0741	0.4819	0.1486	0.093	-0.224	0.273	**32.11**	0.172	0.327	99	101	-0.88416	0.079208

rpl20	0.45	0.13	0.413	0.2179	0.104	-0.157	0.31	48.61	0.265	0.379	113	117	-0.4906	0.08547
clpP	0.471	0.1613	0.4631	0.1231	0.19	-0.087	0.355	52.14	0.224	0.402	183	194	0.154124	0.092784
psbB	0.5161	0.1382	0.3673	0.1729	0.197	-0.062	0.38	46.99	0.249	0.437	481	508	0.109842	0.145669
petB	0.5304	0.1547	0.362	0.1208	0.219	-0.01	0.393	40.42	0.229	0.396	201	217	0.552996	0.138249
petD	0.4733	0.1133	0.4593	0.1301	0.165	-0.128	0.301	42.45	0.199	0.377	166	174	0.513218	0.109195
rpoA	0.4674	0.1571	0.5021	0.177	0.151	-0.152	0.323	48.63	0.242	0.339	322	329	-0.31337	0.091185
rps11	0.4672	0.0902	0.4138	0.1698	0.16	-0.067	0.373	48.23	0.216	0.449	134	138	-0.38406	0.036232
rps8	0.4298	0.1754	0.4808	0.119	0.113	-0.038	0.38	40.77	0.233	0.353	129	134	-0.34179	0.059701
rpl14	0.4554	0.1584	0.4583	0.1463	0.165	-0.046	0.373	48.23	0.237	0.396	118	122	-0.03279	0.040984
rpl16	0.4074	0.1574	0.5	0.1134	0.149	0.015	0.43	40.14	0.219	0.431	128	136	-0.46103	0.080882
rps3	0.3963	0.2134	0.5789	0.0876	0.151	-0.099	0.36	48.27	0.223	0.343	211	218	-0.34358	0.091743
rpl22	0.4712	0.0769	0.534	0.1444	0.128	-0.233	0.272	45.33	0.168	0.354	125	129	-0.27907	0.062016
**rpl2**	0.3922	0.2069	0.4182	0.1859	0.143	-0.093	0.362	**54.24**	0.317	0.442	268	275	-0.65309	0.050909
ycf2	0.4337	0.218	0.3967	0.2627	0.159	-0.118	0.349	52.96	0.351	0.38	2167	2260	-0.45288	0.123894
ndhB	0.4282	0.221	0.434	0.1154	0.163	-0.089	0.353	47.91	0.276	0.374	482	510	0.676667	0.131373
rps7	0.425	0.1333	0.5194	0.1282	0.186	-0.065	0.383	45.95	0.208	0.406	149	155	-0.54516	0.045161
ndhF	0.5272	0.1244	0.4316	0.1409	0.147	-0.174	0.302	44.7	0.202	0.321	709	741	0.572875	0.148448
ccsA	0.4944	0.1124	0.5	0.1183	0.142	-0.243	0.264	43.16	0.176	0.303	296	312	0.648718	0.163462
ndhD	0.4792	0.1366	0.4789	0.1047	0.132	-0.193	0.287	43.77	0.193	0.34	467	500	0.751	0.162
ndhE	0.5517	0.1379	0.4	0.1111	0.172	-0.145	0.32	48.41	0.196	0.32	97	101	0.751485	0.09901
ndhG	0.5093	0.118	0.4135	0.1402	0.132	-0.255	0.24	46.99	0.199	0.33	171	176	1.113636	0.130682
ndhI	0.5185	0.163	0.4455	0.1702	0.192	-0.124	0.344	46.38	0.242	0.358	157	165	-0.07455	0.121212
ndhA	0.471	0.1	0.4844	0.1174	0.127	-0.153	0.298	43.53	0.173	0.338	346	361	0.73518	0.130194
ndhH	0.4787	0.1344	0.5052	0.1423	0.149	-0.144	0.322	47.93	0.207	0.376	367	393	-0.14758	0.114504
ycf1	0.4823	0.1642	0.5514	0.1623	0.157	-0.118	0.356	47.14	0.228	0.29	1808	1879	-0.64396	0.121341

Table S3. List of species used for phylogenetic tree

Family	Plant Name	NCBI Ref. No

Actinidiaceae	*Actinidia kolomikta*	NC_034915
	Actinidia tetramera	NC_031187
	Actinidia polygamya	NC_031186
	Clematoclethra scandens	KX345299
Ebenaceae	*Diospyros kaki*	NC_030789
	Diospyros lotus	NC_030786
	Diospyros oleifera	NC_030787
	Diospyros glaucifolia	NC_030784
Ericaceae	*Arbutus unedo*	JQ067650
	Pyrola rotundifolia	KU833271
	Vaccinium macrocarpon	JQ757046
Lecythidaceae	*Barringtonia fusicarpa*	NC_035701
	Barringtonia racemose	NC_035705
Pentaphylacaceae	*Adinandra angustifolia*	NC_035653
	Adinandra millettii	NC_035678
	Anneslea fragrans	NC_035709
	Pentaphylax euryoides	NC_035710
	Ternstroemia gymnanthera	NC_035706
Primulaceae	*Androsace bulleyana*	NC_034641
	Ardisia polysticta	NC_021121
	Lysimachia coreana	NC_026197
	Maesa montana	KU569490
	Primula chrysochlora	NC_034678
	Primula persimilis	NC_034331
	Primula stenodonta	NC_034677
Sapotaceae	*Pouteria campechiana*	NC_033501
Sladeniaceae	*Sladenia celastrifolia*	NC_035707
Styracaceae	*Bruinsmia polysperma*	NC_030180
	Melliodendron xylocarpum	NC_035712
	Sinojackia xylocarpa	NC_035418
	Styrax grandifloras	NC_030539

Symplocaceae	*Symplocos costaricana*	NC_035708
	Symplocos paniculata	NC_035702
Theaceae	*Polyspora speciosa*	NC_035643
	Pyrenaria diospyricarpa	NC_035704
	Schima wallichii	NC_035546
	Stewartia rostrate	NC_035698
	Camellia yunnanensis	NC_022463
Cornaceae (Outgroup Cornales)	*Diplopanax stachyanthus*	NC_029750
	Nyssa sinensis	KX904873
Hydrangeaceae (Outgroup Cornales)	*Hydrangea luteovenosa*	NC_035662
	Hydrangea serrata	KU140669
